# Supplementary material for: Unique SMYD5 Structure Revealed by AlphaFold Correlates with Its Functional Divergence
Source: Biomolecules. 2022 Jun 3;12(6):783. doi: 10.3390/biom12060783 (PMC9221539; doi:10.3390/biom12060783)
Supplement: Supplementary file 1 [file biomolecules-12-00783-s001.zip › biomolecules-1737681-supplementary.pdf]

Table S1. Statistics of SMYD crystal structures.

PDB	Resolution (Å)	R_{work}	R_{free}	Residue range	Space group	Unit Cell
**SMYD3**						
3MEK	2.1	0.174	0.208	3-425	$P2_12_12_1$	61, 66, 108
3OXL	3.6	0.241	0.283	1-426		55, 101, 117
3PDN	1.7	0.155	0.189	2-428		61, 66, 107
3QWP	1.53	0.174	0.217	3-427		61, 66, 107
5CCL	1.5	0.113	0.164	3-427		59, 65, 106
5CCM	2.3	0.201	0.255	3-425		60, 66, 107
5EX0	2.7	0.178	0.225	1-428		54, 104, 117
5EX3	2.41	0.184	0.223	1-428		54, 105, 118
5HI7	2.15	0.198	0.226	3-428		60, 66, 105
5V37	1.42	0.228	0.279	3-426		61, 66, 107
5XXD	2.31	0.201	0.259	3-425		61, 66, 107
5XXG	2.14	0.185	0.243	3-425		61, 66, 107
5XXJ	1.69	0.202	0.227	3-425		61, 66, 108
5YJO	2.13	0.19	0.232	3-425		61, 66, 108
6IJL	2.35	0.281	0.316	3-425		61, 66, 107
6O9O	1.59	0.172	0.2	3-428		61, 67, 107
6P6K	1.55	0.178	0.214	3-428		61, 66, 106
6P7Z	1.19	0.181	0.198	3-428		61, 66, 107
6PAF	1.24	0.172	0.188	3-428		61, 66, 107
6YUH	1.93	0.186	0.23	3-427		60, 66, 106
7BJ1	1.61	0.172	0.21	3-428		61, 66, 107
3OXF	2.82	0.211	0.261	3-428	$P2_1$	58, 118, 83
3RU0	1.85	0.200	0.216	4-428		58, 118, 83
5HQ8	1.72	0.174	0.201	3-428		53, 118, 85
3OXG	3.41	0.224	0.249	3-426	$P6_1$	103, 103, 112
**SMYD2**						
3TG4	2.0	0.19	0.224	6-432	$P2_12_12_1$	52, 67, 142
3TG5	2.3	0.241	0.263	6-430		53, 72, 121
4WUY	1.63	0.151	0.2	5-433		89, 100, 60
4YND	2.79	0.198	0.253	3-430		53, 71, 119
5ARF	1.92	0.257	0.301	6-430		130, 52, 70
5ARG	1.99	0.23	0.273	6-431		52, 70, 131
6CBX	1.94	0.180	0.223	5-432	$P2_1$	58, 117, 64
6CBY	2.55	0.199	0.258	5-432		58, 118, 65
6MON	2.71	0.210	0.227	5-433	$P4_2$	154, 154, 53
3RIB	2.79	0.246	0.3	7-433	$C2$	195, 57, 97
5V3H	2.69	0.217	0.27	1-432		158, 55, 80
5WCG	2.02	0.17	0.213	2-432		92, 98, 59
3S7B	2.42	0.174	0.198	5-433	$I4$	156, 156, 53
3S7D	2.3	0.184	0.211	5-433		154, 154, 53
3S7F	2.85	0.172	0.201	5-433		156, 156, 53
3S7J	3.04	0.194	0.21	6-433		155, 155, 53
4O6F	2.82	0.175	0.235	3-432		152, 152, 53
5KJK	1.93	0.178	0.203	5-433		156, 156, 52
5KJL	2.7	0.172	0.236	6-433		155, 155, 53
5KJM	2.19	0.176	0.214	5-433		156, 156, 53
5KJN	2.72	0.162	0.225	5-433		155, 155, 52
6N3G	2.43	0.175	0.222	4-433		152, 152, 54

Table S2. PXLXP motifs in SMYD5 interacting proteins.

Protein	UniProt Accession	Number of PXLXP motif
AVL9	Q8NBF6	1
BRD4	O60885	5
DAK	Q3LXA3	0
DCD	P81605	0
FBXL19	Q6PCT2	3
KDM7A	Q6ZMT4	0
MOV10	Q9HCE1	1
NXF1	Q9UBU9	1
PEA15	Q15121	0
TMED132A	Q24JP5	1
TRIM25	Q14258	1
TUB	P50607	0
UBA52	P62987	0
WWOX	Q9NZC7	0
XRCC5	P13010	0
ZNF417	Q8TAU3	0
ZNF430	Q9H8G1	0
ZNF446	Q9NWS9	0
ZNF587	Q96SQ5	0

Table S3. Molecular cloning primers.

**Construct**		**Primer**
hSMYD5(FL)-Myc	F	5′-GTTTTAGGTACCGCCACCATGGCGGCCTCCATG-3'
	R	5′-AATAATCTCGAGGGCTCACAGATCCTCTTCAGAGATGAGTTTCTG CTCACCGCTATCTCCGGTCACATCAGTCATCTC-3'
mSMYD5(FL)-Myc	F	5′-GTTTTAGGTACCGCCACCATGGCGGCCTCCATG-3'
	R	5′-ATATATCTCGAGGGCCTACAGATCCTCTTCAGAGATGAGTTTCTG CTCACCGCTATCTCCGGTCACGTCGGTCATCTC-3'
hSMYD5 (19-418)-Myc	F	5′-TTTTTAGGTACCGCCACCATGGCGCGGGTCTCCGTG-3'
	R	5′-AATAATCTCGAGGGCTCACAGATCCTCTTCAGAGATGAG TTTCT GCTCACCGCTATCTCCGGTCACATCAGTCATCTC-3'
hSMYD5(FL)-GFP	F	5′-TATTTACTCGAGGCCACCATGGCGGCCTCCATGTGC-3'
	R	5′-GCGGCGGGATCCAGCACATCAGTCATCTCATC-3'
mSMYD5(FL)-GFP	F	5′-GTTTTAGGTACCGCCACCATGGCGGCCTCCATG-3'
	R	5′-GAGTCAGGATCCCCCACGTCGGTCATCTC-3'
hSMYD5(1-30)-GFP	F	5′-TCGAGCCACCATGGCGGCCTCCATGTGCGACGTGTTCTCCTTCTG CGTGGGCGTGGCGGGCCGCGCGCGGGTCTCCGTGGAAGTCCGTTTC GTGAGCAGC-3'
	R	5′-GATCGCTGCTCACGAAACGGACTTCCACGGAGACCCGCGCGCG GCCCGCCACGCCCACGCAGAAGGAGAACACGTCGCACATGGAGGC CGCCATGGTGGC-3'
hSMYD5(19-418)-GFP	F	5′-TATTTAGAATTCGCCACCATGGCGCGGGTCTCCGTGGAA-3'
	R	5′-GCGACGGGATCCAGCACATCAGTCATCTCATC-3'
hCOX4 (1-25)-GFP	F	5′-GCCACCATGTTGGCTACCAGGGTATTTAGCCTAGTTGGCAAGCG AGCAATTTCCACCTCTGTGTGT GTACGAGCTCATGAA-3'
	R	5′-GATCTTCATGAGCTCGTACACACACACAGAGGTGGAAATTGCTCGCT TGCCAACTAGGCTAAATACCCTGGTAGCCAACATGGTGGCGTAC-3'
hSMYD1	F	5′-GACTACGGTCTCAAGGTATGACAATAGGGAGGATG-3'
	R	5′-GTACGGATCCTTATTGCTTCTTGTGGAACAG-3'
hSMYD2	F	5′-GATCAAGGTCTCAAGGTATGAGGGCCGAGG-3'
	R	5′-GCAGATCTCGAGTTATCAGTGGCTTTCAATTTCC-3'
hSMYD3	F	5′-GACTACGGTCTCAAGGTATGGAGGCACTGAGGT-3'
	R	5′-GAGAGTGGATCCTTAGGAGGCTCGTATGTTGG-3'
hSMYD5	F	5′-GTATAAGAAGACATAGGTATGGCGGCCTCCATGTGC-3'
	R	5′-CACGGCCTCGAGTTACACATCAGTCATCTCATC-3'

F: forward; R: reverse.

Figure S1

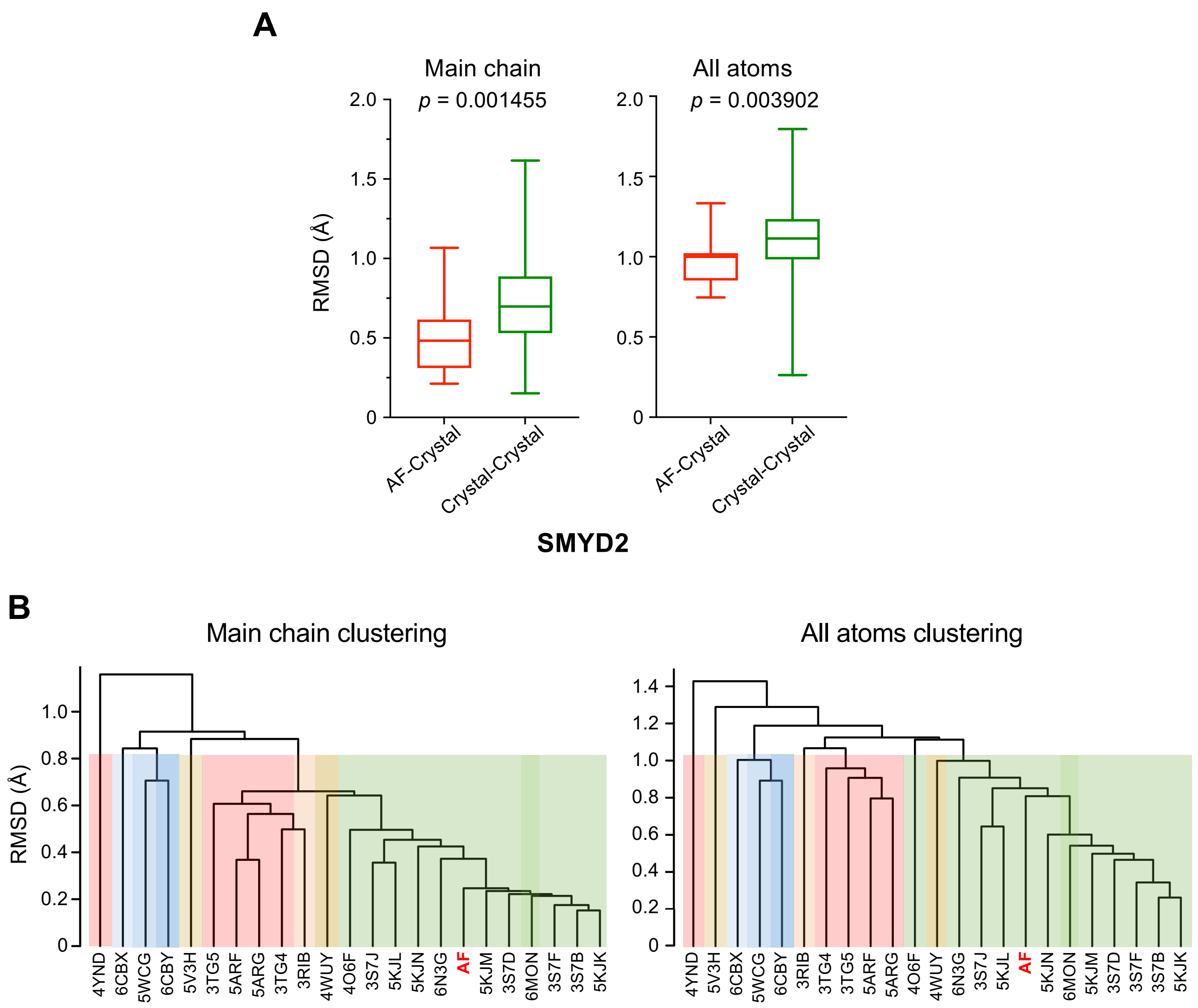

Figure S1. Comparison of AlphaFold and crystal structures of SMYD2 based on RMSD values of the main chain or all atoms. (**A**) Boxplot of RMSD values between AF and crystal structures and between the crystal structures. (**B**) Hierarchical clustering using pairwise RMSD values as dissimilarity measures. Clusters are shaded with the same coloring scheme in Fig. 1B.

Figure S2

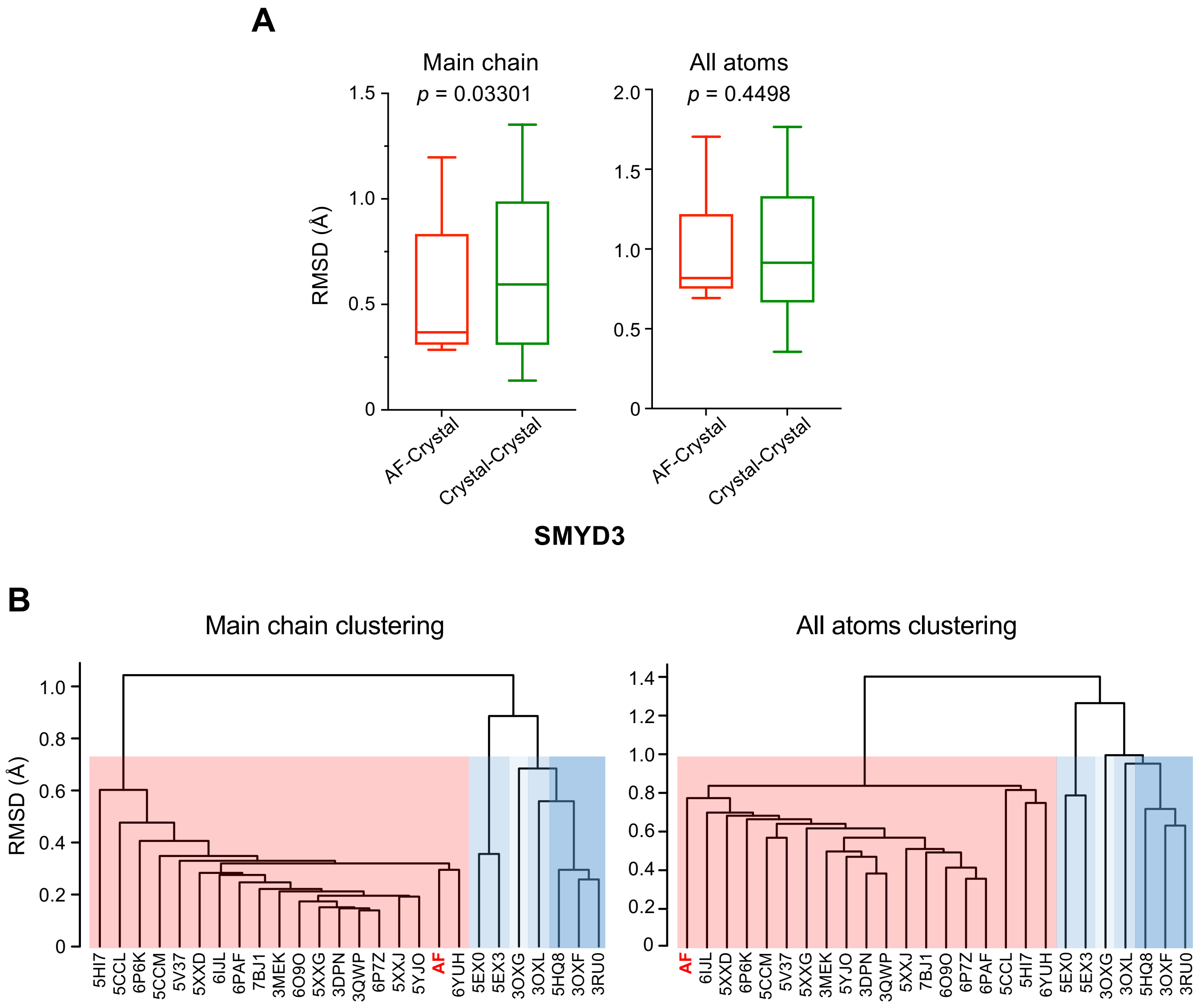

Figure S2. Comparison of AlphaFold and crystal structures of SMYD3 based on RMSD values of the main chain or all atoms. (**A**) Boxplot of RMSD values between AF and crystal structures and between the crystal structures. (**B**) Hierarchical clustering using pairwise RMSD values as dissimilarity measures. Clusters are shaded with the same coloring scheme in Fig. 1E.

Figure S3

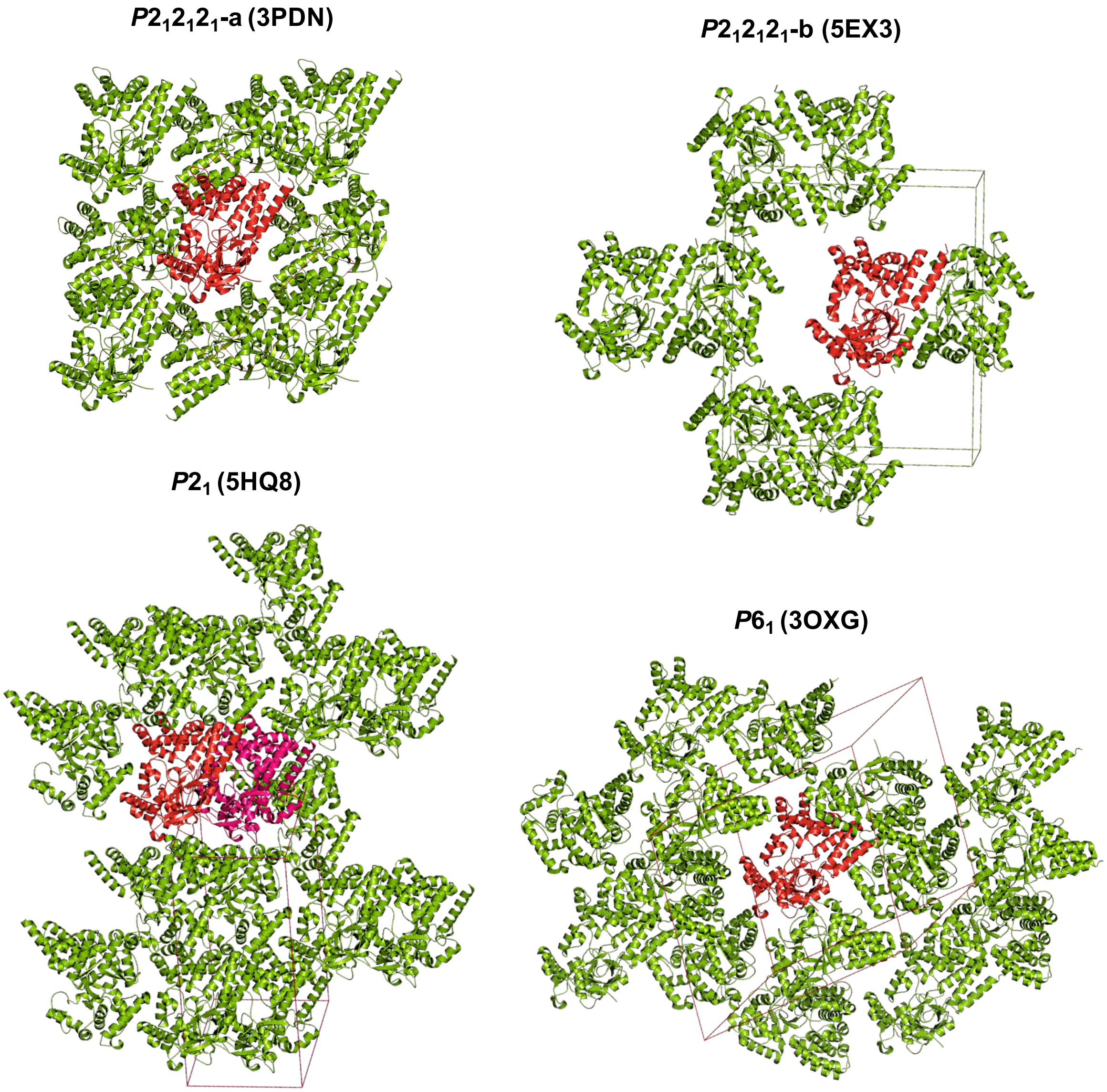

Figure S3. Crystal packing of SMYD3 crystal lattices.

Figure S4

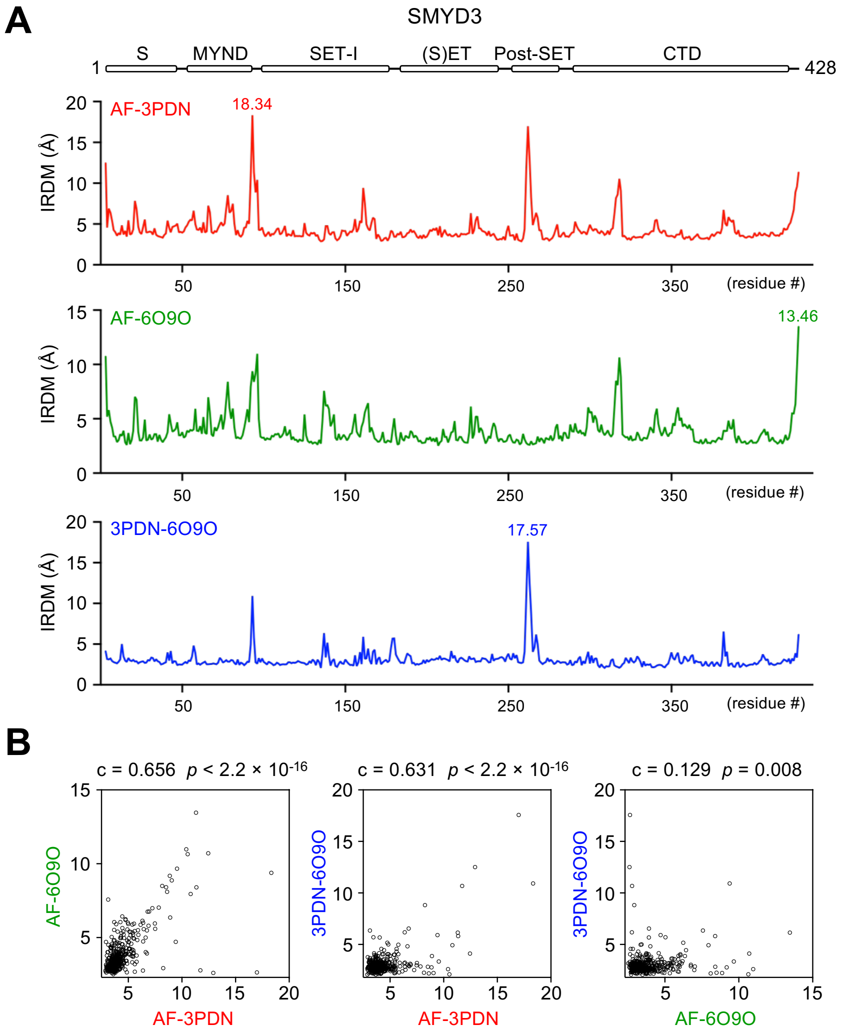

Figure S4. IRDM-based residue-wise structural comparison of SMYD3 AlphaFold and crystal structures. (**A**) A plot of IRDM as a function of residue number between AF and 3PDN (top), between AF and 6O9O (middle), and between 3PDN and 6O9O (bottom). (**B**) Scatter plot of IRDM values between AF–3PDN and AF–6O9O (left), between AF–3PDN and 3PDN–6O9O (middle), and between AF–6O9O and 3PDN–6O9O (right).

Figure S5

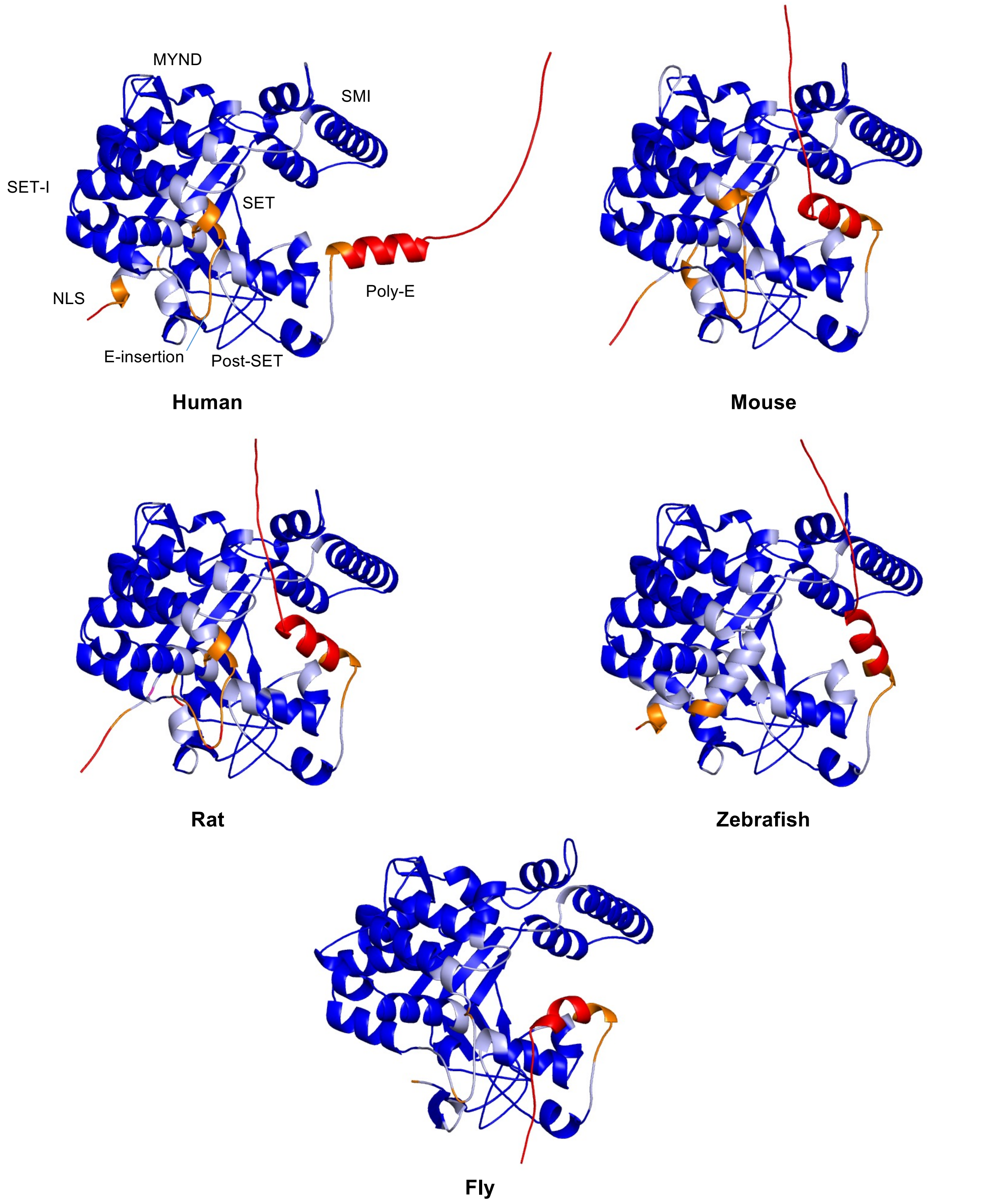

Figure S5. SMYD5 AF structures colored by the AF confidence scores (pLDDT). pLDDT is between 0 and 100. For model confidence, blue: very high (pLDDT > 90); light blue: confident (90 > pLDDT > 70); orange: low (70 > pLDDT > 50); red: very low (pLDDT).

Figure S6

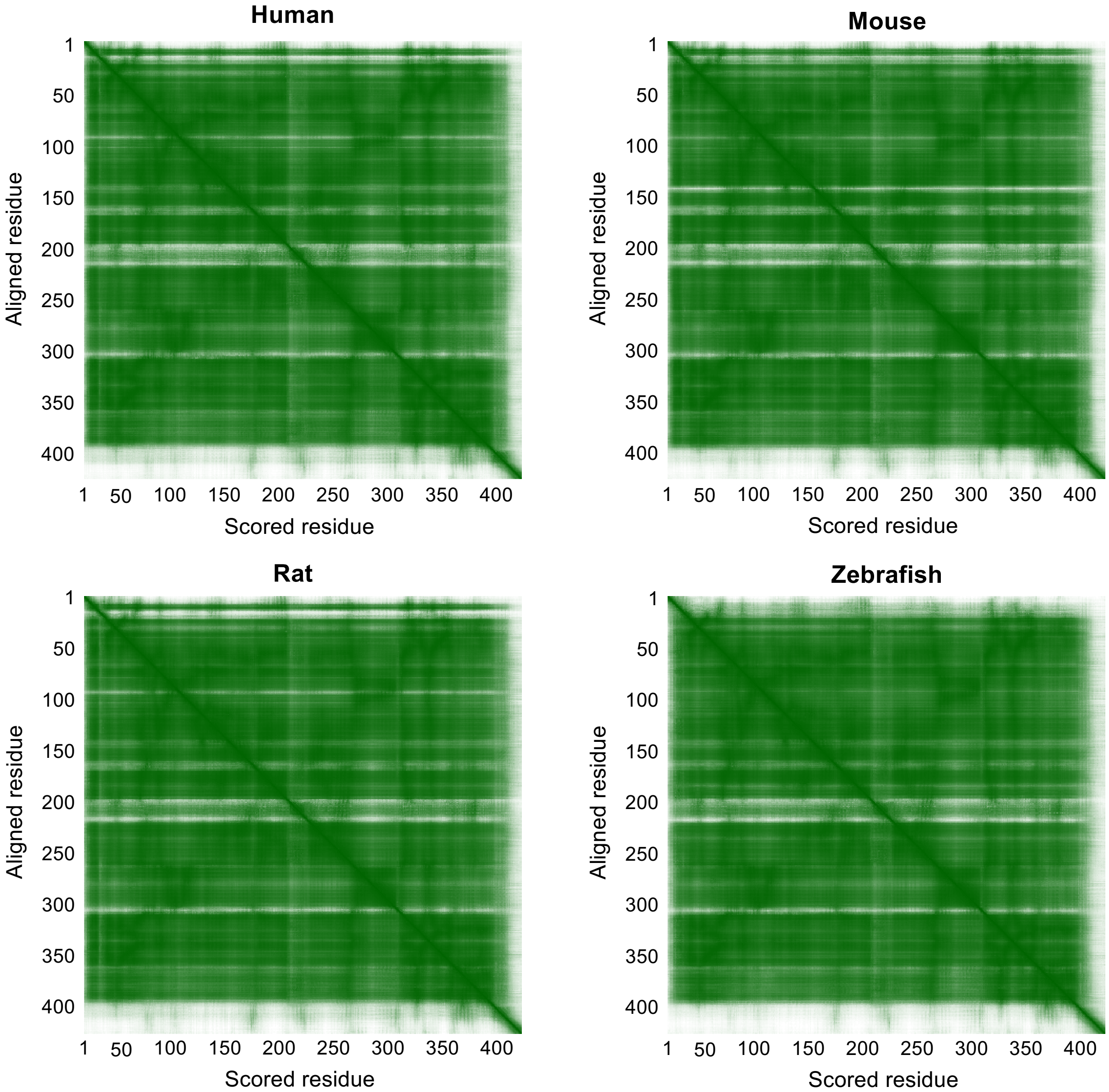

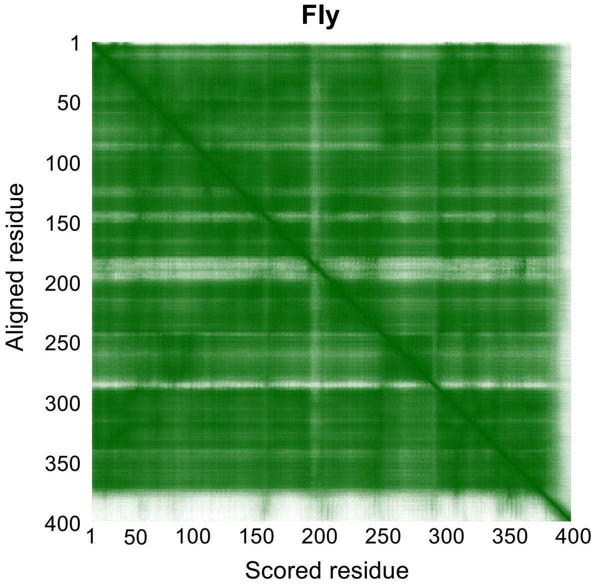

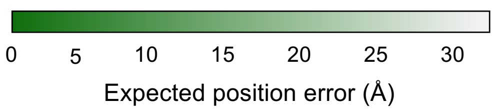

Figure S6. Predicted aligned errors (PAEs) of SMYD5 AF structures.

Figure S7

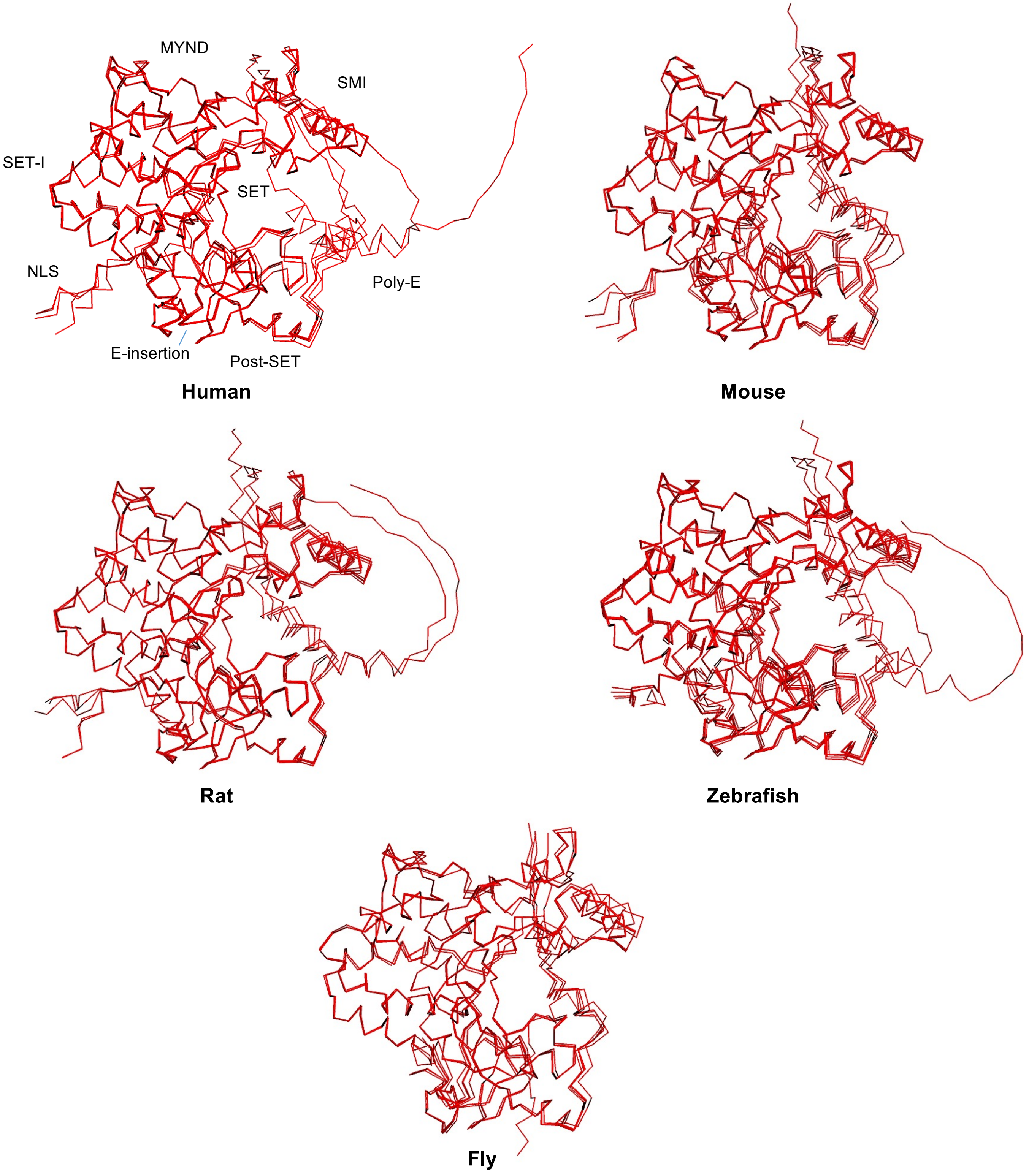

Figure S7. Superposition of five different SMYD5 AF structures generated using different random seeds.

Figure S8

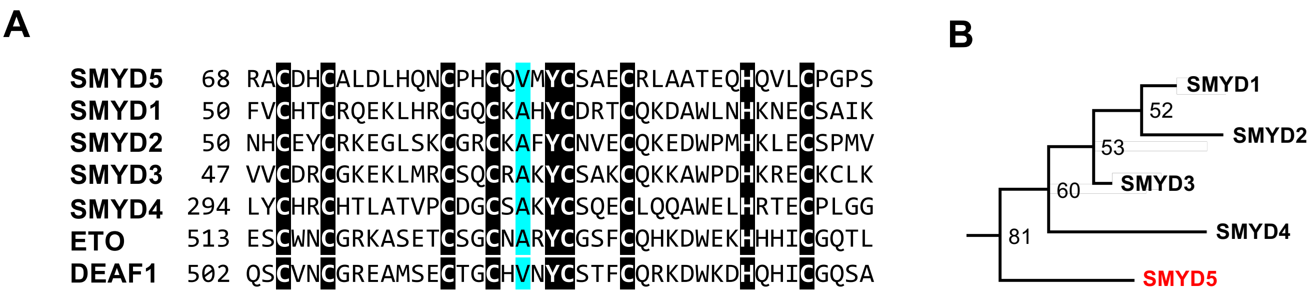

Figure S8. Phylogenetic analysis of MYND domains of human SMYD proteins. (A) Sequence alignment of the MYND domains used for phylogenetic analysis. The MYND domain of ETO or DEAF1 was used as an outgroup to root trees. (B) Phylogenetic tree built by the maximum likelihood algorithm. Bootstrapping values are given for each interior branch. Branch lengths are scaled to the number of substitutions per site.

Figure S9

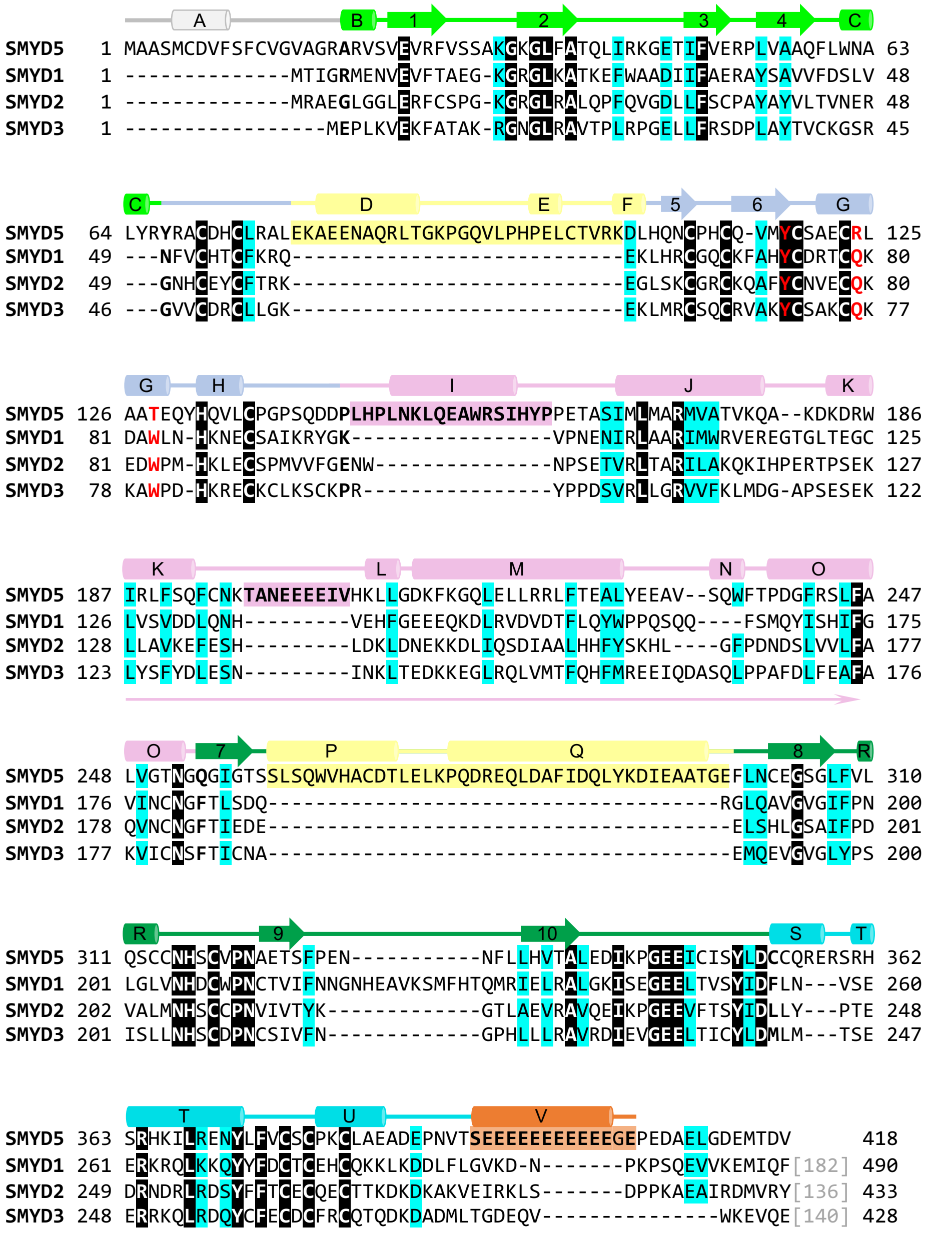

Figure S9. Sequence alignment of human SMYD proteins. Identical residues are shown as white on black, and similar residues appear shaded in cyan. Secondary structures of SMYD5 are indicated above the alignment. Sequence numbering is displayed to the left and right of the sequences.

Figure S10

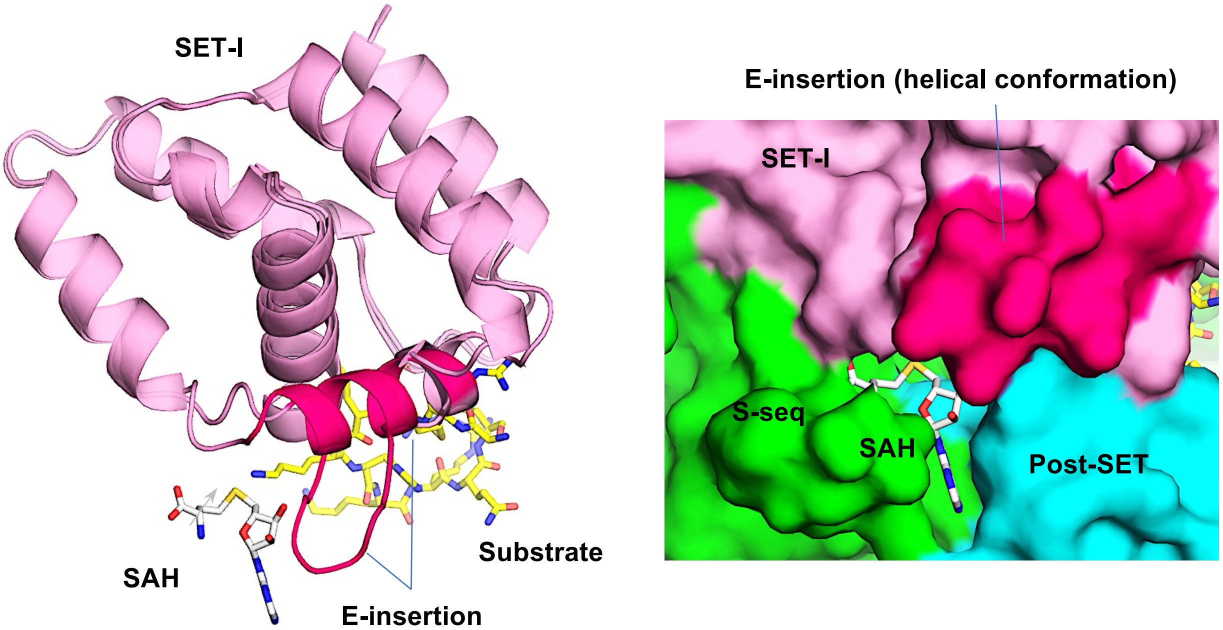

Figure S10. Conformations of E-insertion. (A) Superposition of the helical and loop conformations of the E-insertion. (B) Surface representation shows the location of the helical conformation of the E-insertion relative to the cofactor binding site.

Figure S11

	N-terminal sequence (1-30)	Mito Evidence IMPI*	Mito Evidence IMPI score#	Mito Targeting Seq MitoProt$	DeepMito Prediction&
Human SMYD5	MAASMCDVFSFCVGVAGRARVSVEVRFVSS	Predicted	0.82	0.489	Yes
Human COX4	MLATRVFSLVGKRAISTSVCVRAHESVVKS	Known	1.0	0.981	Yes

Figure S11. Sequence analysis of the N-terminal sequence of SMYD5. The N-terminal sequence was predicted as a mitochondrial targeting signal by MitoMiner 4.0 [45] and DeepMito [46]. Hydrophobic and basic residues are shaded in pink and blue, respectively. *Gene categorization based on the integrated Mitochondrial Protein Index (IMPI) reference set of mitochondrial genes. #IMPI score, ranging from 0 to 1, with 0.8 or above indicating mitochondrial localization. $MitoProt prediction score. &DeepMito predicts SMYD5 localizing to the mitochondrial outer membrane and COX4 to the mitochondrial matrix.
